# Optimized Protocol for the Isolation of Extracellular Vesicles from the Parasitic Worm *Schistosoma mansoni* with Improved Purity, Concentration, and Yield

**DOI:** 10.1155/2022/5473763

**Published:** 2022-04-08

**Authors:** Marije E. Kuipers, Roman I. Koning, Erik Bos, Cornelis H. Hokke, Hermelijn H. Smits, Esther N. M. Nolte-‘t Hoen

**Affiliations:** ^1^Department of Parasitology, LUMC, Leiden, Netherlands; ^2^Department of Biomolecular Health Sciences, Faculty of Veterinary Medicine, Utrecht University, Utrecht, Netherlands; ^3^Department of Cell & Chemical Biology, LUMC, Leiden, Netherlands

## Abstract

In the past decade, the interest in helminth-derived extracellular vesicles (EVs) increased owing to their role in pathogen-host communication. However, the availability of EVs from these parasitic worms is often limited due to the restricted occurrence and culturing possibilities of these organisms. *Schistosoma mansoni* is one of several helminths that have been shown to release EVs affecting the immune response of their host. Further investigation of mechanisms underlying these EV-induced effects warrants separation of EVs from other components of the helminth excretory/secretory products. However, isolation of high-purity EVs often come to the expense of reduced EV yield. We therefore aimed to develop an optimized protocol for isolation of EVs from *S. mansoni* schistosomula and adult worms with respect to purity, concentration, and yield. We tested the use of small (1.7 ml) iodixanol density gradients and demonstrated that this enabled western blot-based analysis of the EV marker protein tetraspanin-2 (TSP-2) in gradient fractions without additional concentration steps. Moreover, the concentration and yield of EVs obtained with small iodixanol gradients were higher compared to medium-sized (4.3 ml) or conventional large-sized (12 ml) gradients. Additionally, we provide evidence that iodixanol is preferred over sucrose as medium for the small density gradients, because EVs in iodixanol gradients reached equilibrium much faster (2 hours) and iodixanol but not sucrose was suitable for purification of schistosomula EVs. Finally, we demonstrate that the small iodixanol gradients were able to separate adult worm EVs from non-EV contaminants such as the blood digestion product hemozoin. Our optimized small iodixanol density gradient allows to simultaneously separate and concentrate EVs while reducing handling time and EV loss and can be applied for EVs from helminths and other limited EV sources.

## 1. Introduction

Extracellular vesicles (EVs) are nanosized, lipid bilayer-enclosed particles containing (glycosylated) proteins, lipids, and RNAs that are released into the extracellular space by virtually all cells and organisms. EVs are widely investigated for their role in communication between cells in various diseases [1–3], in host-pathogen interactions [4, 5], and for their biomarker or therapeutic potential [6–8]. EVs from parasitic worms have emerged as important players in host-pathogen interactions and their role in parasitic diseases warrants extensive investigations [9]. Research on such EVs faces many technical challenges [9–11].

To characterize their molecular composition and function, EVs need to be isolated from biological fluids and separated from large molecular (lipo) protein complexes that overlap in size and/or buoyant density [12]. These purification steps generally reduce EV yield [13, 14], and it has been shown that adsorption of EVs to plastic surfaces, e.g., from (ultra) centrifugation tubes, results in EV loss over time [15]. This loss of EVs could be compensated by increasing the starting material, but this poses an extra challenge when working with limited material, such as parasites. Additionally, the complex life cycle of helminths poses a technical challenge for EV research in this area. The life cycles of many helminths depend on mammalian and intermediate hosts, and one of these helminths is *Schistosoma mansoni*. Obtaining the different schistosome life stages for culture and subsequent EV isolation is challenging due to limited availability of larvae and the restricted amount of parasites obtained from sacrificed infected animals [16]. The schistosomula are transformed cercarial larvae that can only be obtained from an infected intermediate snail host. Adult worms are obtained from perfusion of the portal venous system of mice or hamsters infected with cercarial larvae, and eggs can be isolated from enzymatically digested liver and gut tissue from the infected animals. EVs are released by all three life stages as part of the complex excretory/secretory (E/S) products released by *S. mansoni* [17–19]. Studying their EVs requires separation from other proteins and lipids in these E/S products. E/S from blood feeding parasites, among which is *S. mansoni*, also contains digestion products of blood that the adult worms regurgitate when they are cultured *ex vivo* in serum-free medium [20, 21]. One of these products is hemozoin, a nontoxic, insoluble, and crystallized form of the otherwise toxic heme.

The discovery of (glyco)proteins and RNAs that are specifically enriched in schistosome EVs will accelerate research on how these EVs interact with and manipulate their hosts and on the biomarker potential of these EVs. This kind of research, however, demands the acquisition of sufficient amounts of highly purified EVs. We aimed to develop an optimized method for separating adult worm and schistosomula EVs from non-EV contaminants while keeping the EV yield as high as possible. We propose the use of small-sized iodixanol density gradients to increase concentration and yield of obtained EV isolates while reducing handling time.

## 2. Materials and Methods

### 2.1. Parasite Culture

The life cycle of *S. mansoni* (Puerto Rican strain) was maintained in golden Syrian hamsters (HsdHan-Aura) and *Biomphalaria glabrata* snails as previously described [22]. All hamster experiments were performed in accordance with the Guide for the Care and Use of Laboratory Animals of the Institute for Laboratory Animal Research and have received approval from the university Ethical Review Board (Leiden University Medical Center, Leiden, The Netherlands). Cercariae from shed snails were washed with >25 ml cold DMEM (high glucose with L-glutamine, Lonza, Basel, Switzerland) supplemented with Antibiotic Antimycotic Solution (ABAM, Sigma-Aldrich, St. Louis, MO, USA) in a 30 *μ*m pluriStrainer (pluriSelect, Leipzig, Germany) and subsequently resuspended in 12 ml warm medium (37°C) and transformed to schistosomula by pipetting with a 10 ml serological pipet and incubation at 37°C for 20 minutes. Schistosomula (cercariae without tails) were collected using an orbital shaker and cultured at 37°C and 5% CO_2_ for three days in DMEM + ABAM at 7,500 schistosomula/ml in 25 cm^2^ polystyrene flasks (Greiner Bio-One, Alphen a/d Rijn, The Netherlands).

Obtained adult worms (mixed sex) from perfused hamsters were washed five times or more with >25 ml DMEM + ABAM supplemented with 10 mM HEPES (pH 7.4). Residual hair and tissue, blot clots, and dead worms were removed. Worms were cultured in DMEM + ABAM + HEPES for two days at 37°C and 5% CO_2_ and at 10 worms/ml in 75 cm^2^ polystyrene flasks (Corning, Sigma-Aldrich) with a maximum of 40 ml/flask. Viability of the worms was ensured during and after culture by visual inspection of worm movement and attachment of the worms' sucker to the flask bottom.

### 2.2. Differential Ultracentrifugation (dUC)

Culture medium of the schistosomula and adult worms was collected in 15 ml tubes and 50 ml tubes (Greiner Bio-One), respectively, and centrifuged twice at 200 × *g* and twice at 500 × *g*, all for 10 minutes and at 4°C with low brake (SX4750A rotor and an Allegra X-15R centrifuge) (Beckman Coulter, Brea, CA, USA). After each centrifugation step, supernatants were transferred to a new tube using a serological pipet. The 500 × g supernatants were centrifuged (all in 15 ml tubes) 30 minutes at 5,000 × *g* (SX4750A rotor) and 4°C (max brake). The 5,000 × *g* supernatants were stored at -80°C till further use.

The frozen 5,000 × g supernatants (11-66 mL) were thawed overnight at 4°C, transferred to polypropylene tubes, and ultracentrifuged for 65 minutes 4°C, at 28,000 rpm (~100,000 × g, k-factor 265) in an XE-90 centrifuge using an SW 41 Ti rotor (Beckman Coulter). Subsequently, supernatant was aspirated until the liquid surface reached the conical part of the tube. If this was the final spin, the rest of the supernatant was decanted after which the walls of the tube were wiped dry with a tissue while holding the tube upside down. When a washing step was performed, pellets were resuspended in supernatant remaining in the conical part of the tube, pooled to one tube, topped up with cold PBS (B. Braun, Melsungen, Germany), and spun as before. This washing step was then repeated once more followed by the steps for the final spin. Final EV-enriched pellets were resuspended in PBS or PBS supplemented with 0.2% BSA (Sigma-Aldrich), which was made from a 5% BSA in PBS stock that was cleared from protein aggregates by overnight ultracentrifugation at 100,000 × g. Washed UC pellets for cryo electron microscopy of schistosomula were prepared as described in [23].

### 2.3. Size Exclusion Chromatography (SEC)

The SEC column (qEVoriginal, 70 nm, IZON Science LTD, Christchurch, Aotearoa-New Zealand) was washed with 10 ml PBS at room temperature. Subsequently, 170 *μ*l of ultrafiltrated adult worm culture E/S, corresponding to material from ~100 worms, was loaded onto the column followed by addition of 10 ml PBS, after which 25 consecutive eluted fractions of 500 *μ*l were collected. Fractions were stored at -20°C till preparation for SDS-PAGE.

### 2.4. Density Gradient Ultracentrifugation

UC pellets from 11-30 ml adult worm or 40 ml schistosomula culture supernatants were resuspended in a final volume of 70 *μ*l PBS + 0.2%BSA. For bottom-up iodixanol gradient centrifugation, the resuspended EV pellet was mixed gently with 60% iodixanol (Optiprep, Axis-Shield PoC AS, Oslo, Norway). Blocks of 40%, 30%, and 10% were carefully layered on top using a plastic Pasteur pipet. The volumes used for small, medium, and large gradients are displayed in Table 1a. Iodixanol dilutions were made from a 50% dilution and PBS. For the sucrose gradients, a 2.5 M D(+)-sucrose (Biochemica, PanReac AppliChem, Darmstadt, Germany) stock solution was prepared and from that, 2 M and 0.4 M dilutions were made to prepare 14 linear dilutions as described previously [24]. 70 *μ*l of EV pellet was mixed gently with 320 *μ*l 2.5 M sucrose within the small UC tube. Subsequently, 14 fractions with decreasing sucrose densities (from 1.886 M to 0.4 M sucrose) of 105 *μ*l were carefully added. For top-down centrifugation, the gradients were built with equal iodixanol or sucrose volumes first after which 70 *μ*l EVs suspensions were carefully added on top of the 10% iodixanol or 0.4 M sucrose.

Centrifugation times of large (SW 41 Ti), medium (SW 55 Ti), and small (TLS-55) gradients can be found in Table 1b. Centrifugation time for the small sucrose gradients was either two or 13.5 hours on similar speed as the iodixanol small gradient. All were centrifuged in thin-wall polypropylene tubes, at 4°C with slow acceleration and slow deceleration. SW 41 Ti and SW 55 Ti rotors were used in an XE-90 ultracentrifuge and the TLS-55 rotor in an Optima TLX (Beckman Coulter). Volumes of collected fractions of the iodixanol gradients are mentioned in Table 1b. The volume of sucrose gradient fractions was 155 *μ*l. Collected fractions were kept on ice, vortexed, and 15 *μ*l per fraction was used to measure their refractive index (RI) by a CETI refractometer (Medline Scientific, Chalgrove, UK). Densities were calculated with the formulas 3,35∗RI-3,4665 and 2,6448∗RI-2,5263 for iodixanol and sucrose, respectively.

When comparing the different tube sizes, culture medium of the same worm culture was used and EV pellets were pooled and split evenly before building the gradients and each gradient consisted of EV material from 11 ml medium. For the washing of iodixanol density gradient fractions, two fractions were pooled: 400 *μ*l per pool for the medium gradient and 1,650 *μ*l per pool for the large gradient in SW41 and SW32 sized polypropylene tubes, respectively. Tubes were topped up with cold PBS (diluting the gradient fractions ~22×) supplemented with 0.1% BSA (from 5% BSA stock) or with plain PBS. Samples were centrifuged for 65 minutes, 4°C, at 32,000 rpm in an XE-90 centrifuge (average 126,444 × g and k-factor 203 for SW 41 Ti and average 125,755 × g and k-factor 204 for SW 32 Ti) after which the obtained pellets were resuspended in a volume of 1× sample buffer equal to the pooled small gradient fractions. Samples were stored at -20°C until SDS-PAGE.

### 2.5. Ultrafiltration

Adult worm culture medium from ~1,100 worms was centrifuged once at 200 × g, and the supernatant was concentrated in 3 or 10 kDa filter tubes (Amicon Ultra, Millipore, Merck, Darmstadt, Germany) according to the manufacturer's protocol. E/S in the filters was washed 3 times with PBS and finally concentrated to a volume of 1,870 *μ*l, of which aliquots were stored at -80°C till further use.

Fractions 5-8 with a density 1.21-1.07 g/ml of bottom-up small iodixanol density gradient isolated adult worm EVs (from ~660 worms) were pooled, diluted 1 : 1 with PBS, and transferred into an 0.5 ml 10 kDa centrifugal filter unit (Amicon Ultra, Millipore, Merck) that was precoated with 20 *μ*g trypsin-digested BSA. Iodixanol was removed in 5 centrifugation steps of 10 minutes at 14,000 × g, by which the sample was fully loaded after three steps followed by two additional washing steps with 300-400 *μ*l PBS. The sample was concentrated to 50 *μ*l by a final centrifugation step of 20 minutes. This final sample was collected by brief reverse centrifugation of the filter and directly used for cryo electron microscopy (EM).

### 2.6. Cryo Electron Microscopy (Cryo-EM)

300 mesh EM grids (Quantifoil R2/2, Jena, Germany) were glow-discharged by 0.2 mbar air for two minutes using the glow discharger unit of an EMITECH K950X. Three *μ*l of sample was applied per glow-discharged grid, and the grid was vitrified using an EMGP (Leica, Wetzlar, Germany) at room temperature and 100% humidity. For vitrification, excess sample was removed by blotting for one second to Whatman #1 filter paper directly followed by plunging the grid into liquid ethane (-183°C) after which the grid was stored under liquid nitrogen till further use. Cryo-EM imaging was performed at 120 kV on a Tecnai 12 electron microscope (Thermo Fisher Scientific, Waltham, MA, USA) after mounting the grid in a Gatan 626 cryo-holder. A 4 k × 4 k Eagle camera (Thermo Fisher Scientific) was used to record images with focus between 5-10 *μ*m and an 18,000 × magnification (pixel size 1.2 nm).

### 2.7. Trichloroacetic Acid (TCA) Precipitation

Iodixanol density fractions were mixed with 2% (*w*/*v*) Na-deoxycholate (added 1 : 117), and proteins were precipitated by mixing each fraction with cold 100% (*w*/*v*) trichloroacetic acid (TCA) (added 1 : 10) and a 15 minutes incubation on ice. Eppendorf tubes were centrifuged at maximum speed (16,100 × g) for 10 minutes at 4°C and supernatant was discarded. Eppendorf tubes were spun briefly again to allow removal of all remaining supernatant. Protein pellets were subsequently mixed with 100% ice cold acetone (similar volume as original gradient fraction) and incubated for >10 minutes at -20°C and centrifuged as before. This acetone washing step was repeated after which the samples were dried at room temperature for 5 minutes. Fractions were mixed with 200 *μ*l (large gradient fractions) or 100 *μ*l (medium and small gradient fractions) 1× unreduced sample buffer.

### 2.8. Western Blotting

SEC fractions were thawed and vortexed before mixing with 4× Laemmli sample buffer under nonreducing conditions. Iodixanol and sucrose density fractions were either directly mixed with 4× sample buffer or after pooling and washing by UC. All samples were incubated 3 min 100°C directly after mixing with sample buffer, stored at -20°C, and incubated again at 100°C before loading on the SDS-PAGE gel. All samples were loaded 15 *μ*l per lane, with an exception for the TCA samples of the small and medium gradient fractions, which were loaded 2 *μ*l per lane. Samples and 1.5 *μ*l of ladder (PageRuler Plus, Thermo Fisher Scientific) were separated on 12.5% gels, which were subsequently blotted onto methanol activated PVDF membranes. Membranes were blocked with PBS supplemented with 0.1% (*v*/*v*) Tween-20 and 0.2% (*w*/*v*) gelatin from cold water fish skin (Sigma-Aldrich) and overnight incubated with TSP2-2D6 (mouse IgG, 1 : 2,000) (kind gift from Professor Alex Loukas, James Cook University, Australia) monoclonal antibody. Incubated blots were washed several times with blocking buffer and incubated 45 minutes with Goat-*α*Mouse-IgG-HRP (1 : 10,000, Promega, Leiden, The Netherlands) and washed again extensively. Chemiluminescence substrate (SuperSignal West Pico PLUS, Thermo Fisher Scientific) was applied, and Alliance Q9 (UVITEC, Cambridge, UK) imaged blots were analyzed and signals quantified in Fiji/ImageJ [25].

## 3. Results

### 3.1. Worm EV Isolates Obtained by Differential Ultracentrifugation and Size Exclusion Chromatography Contain Non-EV Contaminants

**Figure 1:**
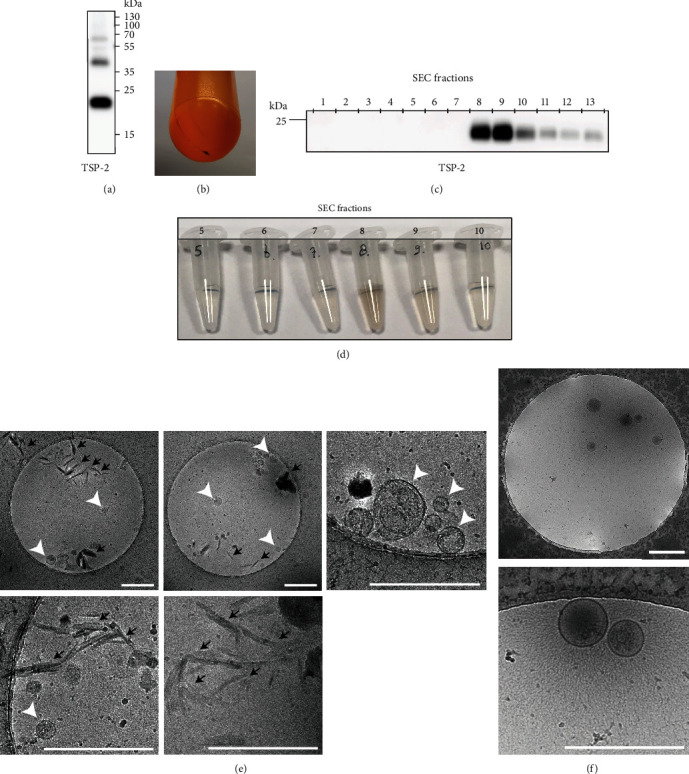
Adult worm EV isolates prepared by differential (ultra)centrifugation (dUC) or size exclusion chromatography (SEC) are contaminated by hemozoin. Culture supernatant of adult worms was subjected to dUC (a, b, and e) or SEC (c, d). (a) Western blot detection of the schistosome EV marker tetraspanin-2 (TSP-2) in 100,000 × g pelleted adult worm EVs. (b) Black 100,000 × g pellet of adult worm EVs indicates the presence of hemozoin. Collected SEC fractions from adult worm E/S released by ~100 worms show similar fractions containing TSP-2 (c) and hemozoin (brown fractions in d). (e) Cryo-EM analysis of washed 100,000 × g pellet from 22 ml adult worm culture shows EVs (white arrowheads) and coisolated hemozoin crystals (black arrows). (f) Cryo-EM analysis shows that pelleted schistosomula EVs from 15 ml culture medium, with characteristic “hair-like” filaments on their surface, do not contain hemozoin crystals. Scale bars (e, f) are 500 nm.

We first tested two size-based separation methods, differential (ultra)centrifugation (dUC) and size exclusion chromatography (SEC), for isolation of EVs released by *Schistosoma* adult worms. As an indicator for the presence of EVs we used western blot analysis for *S. mansoni* tetraspanin-2 (TSP-2), which is one of the most abundant proteins identified in adult worm EVs [26, 27]. Both in the EV-enriched 100,000 x g pellet obtained by dUC and in the EV-enriched SEC fractions, we confirmed the presence of TSP-2 containing schistosome EVs (Figures 1(a) and 1(c)). However, we observed brown coloring of both the UC pellet and the EV-containing SEC fractions (Figures 1(b) and 1(d)). We suspected that this was due to contamination with the blood digestion product hemozoin, which is pigmented and colors the worm gut dark and culture medium light brown. We confirmed the presence of hemozoin crystals in 100,000 × g EV pellets by cryo electron microscopy (cryo-EM) and observed that these crystals overlapped in size with the adult worm EV (Figure 1(e)). This suggests that small hemozoin crystals, and possibly also other non-EV particles [21], sedimented at similar *g*-forces as the worm EVs and could not be separated from EVs based on size differences. The hemozoin contamination was specific for adult worm EV isolates, as EV-enriched dUC pellets from schistosomula did not contain hemozoin (Figure 1(f)). This was expected because schistosomula are transformed from larvae released by snails and cultures of these worms do not contain blood-digestion products. These data indicate that for the design of a protocol that allows purification of EVs from both schistosome life stages, size-based separation methods do not suffice and should be combined with density gradient centrifugation.

### 3.2. The Use of Small Density Gradients Increases EV Concentration and Yield

**Figure 2:**
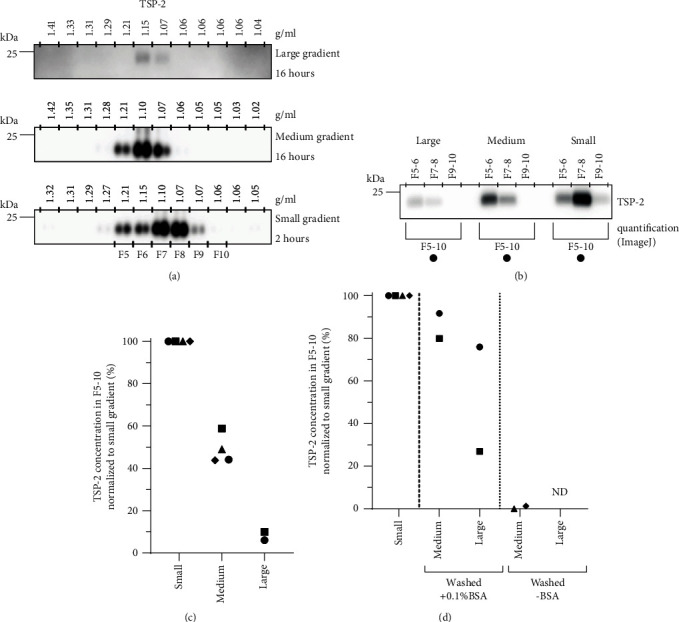
Comparative detection of adult worm EVs isolated with three different sizes iodixanol density gradients. (a) Western blot detection of tetraspanin-2 (TSP-2) in TCA precipitated protein isolates of fractions from adult worm EVs floated bottom-up into large (12 ml), medium (4.3 ml), or small (1.7 ml) iodixanol gradients. (b) Equal 100,000 × g EV-enriched pellets of the same adult worm cultures were loaded in the three differently sized gradients. EV-containing fractions were pooled and directly mixed with sample buffer for SDS-PAGE to compare TSP-2 detection by western blot. (c) Quantification of TSP-2 band intensities of western blots in (b). (d) EVs in pooled fractions of medium and large gradients were diluted in PBS with or without 0.1%BSA, repelleted by UC, and concentrated to equal volumes as the small gradient fractions. TSP-2 levels in concentrated EV isolates from medium- and large-sized gradients were compared to EV isolates taken directly from the small density gradient. TSP-2 western blot and quantification were performed as in (b). Data in (a) and (b) is representative for 2-4 independent experiments. Each symbol in (c) and (d) corresponds to an independent experiment.

When separation based on size is insufficient, EVs can be further purified using density gradient centrifugation [12, 14, 28]. An unwanted consequence of conventional density gradient separation, however, is the dilution of EVs in relatively large volumes of gradient medium. As a consequence, additional concentrating steps are required for further analysis of the EVs. This can be achieved by ultracentrifugation, after pooling the EV-containing density gradient fractions in a larger UC tube, dilution with PBS, and repelleting by UC [28]. This step can cause loss of EVs via adsorption to tube walls and handling time, though the addition of BSA to the PBS may reduce some of the loss [15]. Because loss of EVs is particularly undesirable when working with limited worm-derived material, we aimed to optimize density gradient-based EV isolation for low-input material. We hypothesized that using a small volume density gradient in a small tube will reduce EV loss by decreasing the plastic surface area to which EVs may be adsorbed [15]. In addition, the shorter centrifugation time needed for EVs to reach their equilibrium density in these small tubes reduces exposure time of EVs to the plastic surfaces. Finally, a small gradient introduces a concentrating factor of the EVs as fraction volumes will be smaller. We compared EV isolation efficiency in conventional large-sized (12 ml) and medium-sized (4.3 ml) gradients to a small-sized (1.7 ml) gradient. All three iodixanol gradients were built according to a similar set-up (see Table 1) but the large and medium gradients were centrifuged for >16 hours [28], and the small gradient was centrifuged for two hours [29]. These centrifugation times were sufficient for EVs to float to the ~1.07-1.10 g/ml density fractions, as based on western blot detection of schistosome TSP-2 (Figure 2(a)).

Next, we assessed whether the smaller gradient size reduced adult worm EV loss and enabled western blot-based analysis of EV proteins in gradient fractions without additional concentration steps. To be able to accurately compare EV concentrations within the gradient fractions, adult worm EVs from the same worm culture were equally divided over the three differently sized gradients. After centrifugation, 12 equal volume fractions were collected from each gradient and two consecutive fractions were pooled to allow all EV containing fractions (F5-F10) from the three different gradients to be loaded on the same SDS-page gel. First, the pools of gradient fractions were directly mixed with sample buffer, subjected to SDS-PAGE, and analyzed by western blotting for the presence of TSP-2 (Figure 2(b)). We observed that the concentration in the fractions of the medium and large-sized gradients was 41-56% and 90-94% lower compared to the small gradients, respectively (Figure 2(c)). Thus, the small gradient allows western blot analysis of EV proteins without the need to concentrate the density fractions before gel loading.

Next, we tested the effect of concentration steps on EV recovery from fractions of the medium and large gradients. Based on previous indications that this loss could be reduced in the presence of BSA, the EV containing density fractions were diluted in PBS or PBS supplemented with BSA prior to repelleting of EVs by UC. EV pellets were resuspended in the same volume as the pooled fractions from the small gradient. Western blotting of TSP-2 showed that concentrating the pooled fractions in the presence of BSA resulted in 8-20% lower concentration of EVs from the medium gradient and 24-73% lower concentration for the EVs from the large gradient, as compared to the small gradient fractions (Figure 2(d)). Concentration in the absence of BSA led to an almost complete loss of EVs. Thus, the small gradient is superior over the other sized gradients in EV concentration and yield while the handling time is reduced.

### 3.3. Iodixanol but Not Sucrose Density Gradients Are Suitable for Purification of Adult Worm and Schistosomula EVs

**Figure 3:**
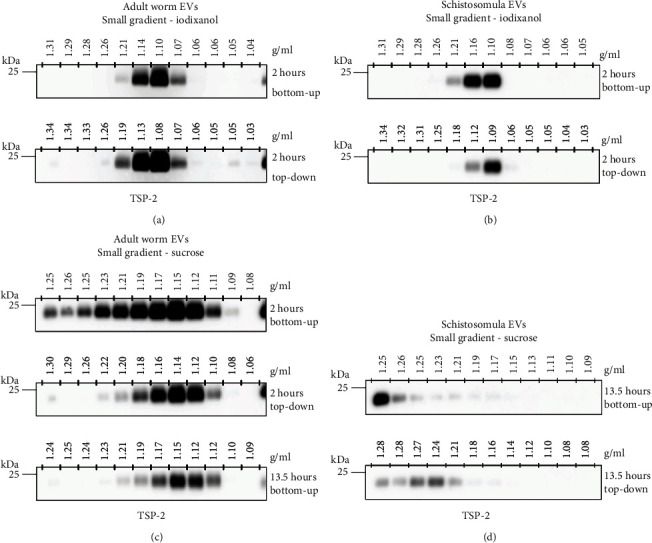
Iodixanol gradients are preferred over sucrose gradients for purification of adult worm and schistosomula EVs. EV-enriched UC pellets of adult worm EVs (a) or schistosomula EVs (b) were loaded at the bottom (bottom-up) or on top (top-down) of a small iodixanol density gradient and centrifuged for two hours. Western blot analysis of collected density fractions for the EV marker TSP-2 shows that both EV types reached equilibrium densities in the small gradients. (c) Adult worm EVs were loaded at the bottom or top of small sucrose density gradients. Shown are western blot detections of TSP-2 in fractions collected from two and 13.5 hours centrifuged sucrose density gradients. (d) Top-down and bottom-up sucrose gradients were used to purify schistosomula EVs. Shown are western blot detections of TSP-2 in fractions collected from 13.5 hours centrifuged gradients. Gradients were loaded with material from 11 or 10 ml culture medium from the adult worms or schistosomula, respectively. Western blots shown are representative for 2-3 independent experiments.

We aimed to optimize an EV purification protocol suitable for EVs of both adult worms and schistosomula. To verify whether both types of EVs reached equilibrium density within 2 hours of centrifugation in these small gradients, we compared loading the gradients on top of the EV-enriched pellets (EVs floating “bottom-up”) to loading the EVs on top of the gradients (EVs floating “top-down”). TSP-2 detection in collected fractions showed that both schistosomula and adult worm EVs concentrated in fractions with densities characteristic for EVs (1.08-1.16 g/ml) and that this was similar in bottom-up or top-down loaded gradients (Figures 3(a) and 3(b)), indicating that equilibrium densities were reached.

Another widely used density gradient medium is sucrose, and we additionally tested whether EVs from both schistosome life stages could also be isolated by a small sucrose gradient. However, two hours of centrifugation was not sufficient for the adult worm EVs to reach equilibrium density in bottom-up sucrose gradients (Figure 3(c)). Under these conditions, part of the TSP-2 remained in the fractions with higher densities, suggesting that the EVs needed more time to reach the lower densities. Indeed, 13.5 hours centrifugation of the bottom-up gradient was needed for the adult worm EVs to reach the 1.12-1.16 g/ml density fractions (Figure 3(c)). In contrast to the EVs from the adult worms, sucrose gradients were less effective in purification of schistosomula EVs. In both bottom-up and top-down sucrose gradients, the schistosomula EVs ended up in fractions with higher densities (between 1.21 and 1.28 g/ml) compared to iodixanol gradients (Figure 3(d)). This is undesirable, since various non-EV contaminants also remain in the bottom fractions of gradients. These data suggest that the small iodixanol gradients are most optimal and time-efficient for purifying EVs from both life stages of *S. mansoni*.

### 3.4. Small Iodixanol Density Gradients Separate EVs from Non-EV Contaminants

**Figure 4:**
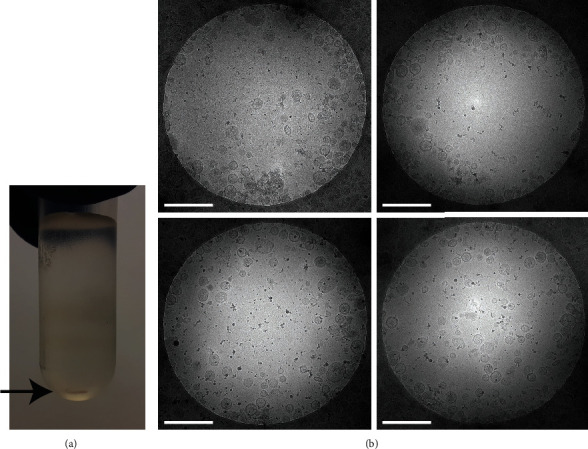
Small iodixanol gradient separates adult worm EVs from non-EV contaminants. Adult worm EV-enriched UC pellets were overlaid with a small (1.7 ml) iodixanol density gradient and centrifuged for two hours. (a) The gradient after centrifugation shows hemozoin (black arrow) in the bottom of the tube. (b) Cryo-EM analysis of washed iodixanol gradient fractions with density 1.07-1.21 g/ml shows that EV-enriched fractions of the small gradients are devoid of hemozoin crystals. Scale bars are 500 nm.

Finally, we verified whether the small iodixanol gradients allowed separation of EVs from non-EV contaminants (Figure 1). We selected hemozoin as detectable example for non-EV contaminants, since markers for other protein contaminants are currently lacking for *Schistosoma* spp. UC pellets of adult worm culture supernatant were overlaid with the optimized small iodixanol density gradient. After two hours centrifugation, we observed a black substance in the bottom of the gradient, suggesting the presence of hemozoin (Figure 4(a)). Cryo-EM analysis of EV-containing fractions (1.07-1.21 g/ml) confirmed the absence of hemozoin among the high number of adult worm EVs in these gradient fractions (Figure 4(b)). These data indicate that the small iodixanol density gradient allowed successful separation of EVs from non-EV particles such as hemozoin. We propose that small-sized (1.7 ml) iodixanol density gradients can be used to simultaneously separate and concentrate EVs from limited sources, such as helminths, to increase EV yield while reducing handling time.

## 4. Discussion

Each EV source has its own challenge for obtaining a high-quality EV preparation with sufficient material for downstream analysis, and these challenges often include the separation of EVs from non-EV contaminants [30]. For *Schistosoma mansoni* parasites, these non-EV contaminants include many released E/S components [17, 18] and for adult worms also the blood-digestion product hemozoin [31]. Both non-EV E/S components and hemozoin are unwanted in EV-preparations, especially when studying host-pathogen interactions as they can affect host immune responses [32, 33]. Additional challenges for studying EVs from *S. mansoni* are imposed by the complex life cycle of this parasite. Not only do the different life stages of the parasite release EVs with different characteristics (Figures 1(e) and 1(f) and [23]), there is also limited availability of each life stage, thus restricting the material to isolate EVs from. This latter restriction makes any loss of EVs during the isolation process highly undesirable. Here, we propose the use of small iodixanol density gradients to increase EV yield compared to larger-sized gradients for separating EVs from non-EV contaminants. This protocol is applicable to EVs from the different *S. mansoni* life stages and EVs from other limited sources.

Methods that separate structures based on size, such as dUC and SEC, were not sufficient to separate adult worm EVs from contaminating particles such as hemozoin crystals (Figure 1). Previously, SEC was proposed as a promising method for helminth EVs [11]. In that study, dUC and SEC were compared for isolation of EVs from *Fasciola hepatica*. Since *F. hepatica* lives in the bile duct and consumes local cells and bile instead of red blood cells for survival, there will be no hemozoin among the non-EV contaminants. However, SEC often dilutes the sample and EVs may be lost upon subsequent concentration steps, which would be unwanted for limited EV sources. Furthermore, a combination of size- and density-based separation methods is preferred to prepare EV populations to the highest purity [14, 28, 30, 34].

Both sucrose and iodixanol are used as gradient media for density-based isolation of EVs. Studies comparing iodixanol and sucrose density gradients for isolation of mammalian EVs showed no clear differences in densities the EVs ended up in [35, 36]. We here provide evidence that the type of gradient medium can strongly influence in which density fraction the *S. mansoni* EVs are concentrated and that this differs between EVs from the different parasite life stages. Our data show that the EVs from schistosomula, in contrast to EVs from adult worms, did not float to the characteristic EV density fractions (~1.10-1.17 g/ml) in sucrose gradients (Figure 3(d)). Yet, these two types of EVs floated to similar density fractions in the iodixanol gradients. The preferential localization of schistosomula EVs in high sucrose densities may possibly be linked to the unique filamentous structures on the schistosomula EV surface of which the actual molecular structure remains unknown [23]. We speculate that these structures contain glycans and/or mucins that interact with the sucrose, thereby influencing their localization in the gradient. There are several advantages of iodixanol over sucrose gradients for the purification of EVs. Iodixanol is isosmotic and isotonic, which is better to preserve EV structure and function. Moreover, iodixanol is far less toxic to cells than sucrose and therefore more suitable to study EV function. Our data substantiates the advantages of iodixanol over sucrose since iodixanol gradients allowed purification of EVs released by both *S. mansoni* life stages and allowed EVs to reach equilibrium density within shorter centrifugation times.

To our knowledge, this is the first report on utilizing small gradients for EV isolation, even though such gradients have been used for isolation of (multi)-protein complexes [29, 37, 38]. In these smaller gradients, EVs reach their equilibrium density after shorter centrifugation times than in conventional large volume gradients, which reduces handling time. Moreover, EVs are concentrated in a smaller volume, which makes it more feasible to directly subject density fractions to western blot analysis without additional concentration steps that may cause EV loss [15]. Another possible benefit of concentrated EV in small volume iodixanol density fractions is that these fractions could be directly added to cells, but this has not yet been studied. Though iodixanol is nontoxic, potential side-effects on cell functions would need to be controlled for. Of note, our proposed small iodixanol gradient was optimized for isolation of schistosome EVs. The exact construction of the gradient may need to be adjusted for optimal separation of EVs from other sources. Therefore, we highly encourage researchers to adjust their own established gradients to smaller-sized tubes.

EV research in the last decade taught us that choosing an appropriate EV isolation method is guided by both the research question and the type of source material. Our optimized protocol increases the concentration and yield of highly purified EVs from *S. mansoni* life stages. This may accelerate research and discoveries in the field including EV-induced immune-modulatory mechanisms and EV-associated biomarkers.

## Figures and Tables

**(a):** 

	Rotor type	Gradient build (iodixanol)				
		EV sample (*μ*L)	60% (*μ*L)	40% (*μ*L)	30% (*μ*L)	10% (*μ*L)
Large	SW 41 Ti	70	3,200	1,600	1,600	5,500
Medium	SW 55 Ti	70	1,100	550	550	1,980
Small	TLS-55	70	440	220	220	792

**(b):** 

Rotor type	Rpm	Average g-force	k-Factor	Centrifugation time (h)	Total gradient volume (*μ*L)	Fraction volume (*μ*L) (12×)
SW 41 Ti	37,000	169,044	152	>16	11,970	997
SW 55 Ti	42,000	169,639	82	>15	4,250	354
TLS-55	50,000	166,180	60	2	1,742	145

## Data Availability

N.A. This manuscript does not include omics-based data that can be archived in public databases. *EV-TRACK*. We have submitted all relevant data of our experiments to the EV-TRACK knowledgebase (EV-TRACK ID: EV220119) [39].
